# Clinical Profile of Neonates Admitted with Sepsis to Neonatal Intensive Care Unit of Jimma Medical Center, A Tertiary Hospital in Ethiopia

**DOI:** 10.4314/ejhs.v31i3.5

**Published:** 2021-05

**Authors:** Melkamu Berhane, Netsanet Workneh Gidi, Beza Eshetu, Mulatu Gashaw, Getnet Tesfaw, Andreas Wieser, Guro K Bårnes, Guenter Froeschl, Solomon Ali, Esayas Kebede Gudina

**Affiliations:** 1 Department of Pediatrics and Child Health, Jimma University; 2 Department of Medical Laboratory Sciences, Jimma University; 3 Max von Pettenkofer-Institute, Ludwig-Maximilians-Universität (LMU), Munich, Germany; 4 Division of Infectious Diseases and Tropical Medicine, Ludwig-Maximilians-Universität (LMU), Munich, Germany; 5 Department of Microbiology, St. Paul Hospital Millennium Medical College; 6 German Center for Infection Research (DZIF), Partner Site Munich, Germany; 7 Department of Internal Medicine, Jimma University; 8 Division for Infection Control and Environmental Health, Norwegian Institute of Public Health, Oslo, Norway; 9 Innlandet Hospital Trust, Division Gjøvik-Lillehammer, Gjøvik, Norway

**Keywords:** Neonatal mortality, neonatal sepsis, isolates, blood culture, Ethiopia

## Abstract

**Background:**

Globally, over 3 million newborn die each year, one million of these attributed to infections. The objective of this study was to determine the etiologies and clinical characteristics of sepsis in neonates admitted to intensive care unit of a tertiary hospital in Ethiopia.

**Methods:**

A longitudinal hospital based cohort study was conducted from April 1 to October 31, 2018 at the neonatal intensive care unit of Jimma Medical Center, southwest Ethiopia. Diagnosis of sepsis was established using the World Health Organization's case definition. Structured questionnaires and case specific recording formats were used to capture the relevant data. Venous blood and cerebrospinal fluid from neonates suspected to have sepsis were collected.

**Results:**

Out of 304 neonates enrolled in the study, 195 (64.1%) had clinical evidence for sepsis, majority (84.1%; 164/195) of them having early onset neonatal sepsis. The three most frequent presenting signs and symptoms were fast breathing (64.6%; 122/195), fever (48.1%; 91/195) and altered feeding (39.0%; 76/195). Etiologic agents were detected from the blood culture of 61.2% (115/195) neonates. Bacterial pathogens contributed for 94.8% (109/115); the rest being fungal etiologies. Coagulase negative staphylococci (25.7%; 28/109), Staphylococcus aureus (22.1%; 24/109) and Klebsiella species (16.5%; 18/109) were the most commonly isolated bacteria.

**Conclusion:**

Majority of the neonates had early onset neonatal sepsis. The major etiologies isolated in our study markedly deviate from the usual organisms causing neonatal sepsis. Multicentre study and continuous surveillance are essential to tackle the current challenge to reduce neonatal mortality due to sepsis in Ethiopia.

## Introduction

In 2018, 5.3 million children died before reaching their fifth birthday of which 2.5 million (47%) died in the first month of life. Sub-Saharan Africa had the highest under five mortality rate, with a neonatal mortality rate of 28 deaths per 1,000 live births. A significant proportion of neonatal mortality is attributable to neonatal infections (1,2). In Ethiopia, the current neonatal mortality rate is 30 per 1000 live births and neonatal infections is one of the top three causes of neonatal mortality (3). Besides increased mortality, neonatal sepsis predisposes to several neuro-developmental complications (4–6).

Diagnosis and management of neonatal sepsis are among the greatest challenges health workers face. Some of the challenges are lack of specific signs and symptoms and unavailability of the necessary laboratory investigations, particularly in developing countries (7,8). This necessitates the initiation of empirical antibiotic therapy till sepsis is either ruled out or confirmed and until specific organisms are isolated. The ever changing patterns of the etiologic agents of sepsis and the dramatically increasing rate of multidrug resistant organisms are also additional challenges on the use of these empiric treatment regimens, delaying the effective treatment of these infections (8,9). Ethiopia is not an exception to these and similar challenges and practices are encountered in many of the health facilities in the country.

Neonatal sepsis is usually classified into early onset neonatal sepsis (EONS) if it occurs in the first 7 days of life and late onset neonatal sepsis (LONS) if it occurs between 7 and 28 days of life. Huge variations are observed in the etiology of sepsis depending on host and environmental factors. Etiological data from low and middle- income countries (LMICs), are very limited, even if some studies conducted in these regions have demonstrated the most common causative agents of neonatal sepsis to be *S. aureus, Escherichia coli* and *Klebsiella spp.* (10–14).

In Ethiopia, only few studies have been published on neonatal sepsis which have indicated the common etiologies to be *S. aureus*, *Coagulase negative staphylococci (CONS)*, *Klebsiella spp* and *E. Coli* (15,16), necessitating further studies to be conducted to characterise the clinical findings and etiologies of neonatal sepsis in the local context. Hence, this study was done with the aim of describing the etiology, clinical characteristics and outcome of neonates admitted to Jimma Medical Center (JMC) with neonatal sepsis. The current study is a sub-study of a large study conducted to determine the magnitude, clinical characteristics, etiologies and antimicrobial susceptibility pattern of the isolates of neonatal sepsis. A separate article on the antimicrobial susceptibility of the isolates will be prepared

## Methods

**Study settings**: The study was conducted at the neonatal intensive care unit (NICU) of JMC, a tertiary hospital in Southwest Ethiopia.

**Study design and period**: A longitudinal hospital based cohort study comparing neonates with signs/symptoms of sepsis to those without signs/symptoms of sepsis was conducted from April 1 to October 31, 2018. In addition, in the subgroup of neonates with signs/symptoms of sepsis, we conducted a descriptive microbiological sub-study that is highlighting the spectrum of infectious agents in neonates with signs/symptoms of sepsis.

**Selection of study participants:** Included in this study were newborns younger than 28 days admitted to the NICU during the study period, enrolled consecutively after their parents or care givers gave informed consent. After enrolment, the neonates were followed until discharge to assess the discharge outcome.

**Data collection procedures:** Patients were categorized on admission into those with clinical diagnosis of sepsis and those without. Case specific recording format was used to capture relevant variables. Both groups of patients were followed according to the protocols of the unit. Neonates with no sepsis on admission who developed signs and symptoms of sepsis after admission were later categorized with the group with sepsis.

The neonates were evaluated and treated by the treating physicians as per the standard protocol at the hospital. Clinical diagnosis of sepsis was made based on the World Health Organization's (WHO) criteria of presence of one or more of the following symptoms: temperature instability (>38 or <35.5°C), tachypnea (≥60breaths/minute), poor feeding/unable to feed, respiratory distress, convulsions, decreased movement or no movement at all (2). Additionally, the presence of risk factors for the development of sepsis was also used to support the diagnosis. Clinical data were collected by trained nurses and physicians working in the NICU (the nurses collected the relevant data and the physicians verified the data collected by the nurses); blood samples were collected by the nurses and cerebrospinal fluid (CSF) by the treating physicians.

**Specimen collection and processing:** In the subgroup of neonates suspected to have sepsis, 1–3ml venous blood was collected and inoculated into aerobic BACTEC bottles (BACTEC Peds Plus/F medium, Becton Dickinson, USA) which were then incubated in BACTEC FX40 automated machine. Samples flagged as positive on BACTEC were subcultured onto Columbia 5% sheep blood, MacConkey, and Chocolate agar (Oxoid, Basingstoke, United Kingdom). Additionally, 2–3ml of CSF was collected under aseptic conditions for analysis and culture. Isolation and identification of bacterial pathogens was performed according to standard microbiological techniques (17). In the subgroup of neonates without clinical suspicion of sepsis, no analysis of microbiological specimen was performed.

**Data processing, analysis and interpretation:** Data was entered into epidata version 3.1 and then exported to and analyzed with SPSS version 20.0. Descriptive statistics like frequency and proportion was carried out to describe the data and results are presented as narrations and using tables and figures.

**Ethical considerations:** Ethical clearance was obtained from Institutional Review Board of Jimma University Institute of Health in Ethiopia (IHRPGD/274/2018) and The Ethics Committee at the Medical Faculty of Ludwig-Maximilians-Universität of Munich, Germany. Written informed consent was obtained from parents or care takers. All study procedures were conducted as per the guidelines of good clinical practices. All laboratory results were timely communicated with the treating physicians so that the treatment of the neonates could be adjusted accordingly.

## Results

**Background characteristics of the neonates:** A total of 304 neonates, 57.9% (176) being male, were enrolled in the study. About 63.0% (188) of them were delivered through spontaneous vaginal delivery. Most of the neonates (258, 86.3%) were younger than 7 days at admission. Only 48.0% (146) of them had their weight determined at birth, 75 (51.4%) being low birth weight (< 2500 g). Gestational age was determined by the New Ballard Score (NBS) for 65.5% (199/304) of the neonates, 41.7% (83/199) being preterm. One fifth (63/304, 20.7%) of the neonates were resuscitated at birth (Table 1). There was no any significant association seen between these variables and blood culture result.

**Table 1:** Background characteristics of neonates admitted to neonatal intensive care unit of JMC

Variable	Category	Total	Sepsis, N (%)	No sepsis, N (%)
Sex (N=304)	Male	176	124 (70.5)	52 (29.5)
	Female	121	69 (57.0)	52 (43.0)
Birth weight (N=146)	<1500	25	7 (28.0)	18 (72.0)
	1500 – 2499	50	16 (32.0)	34 (68.0)
	≥2500	71	48 (67.6)	23 (32.4)
Admission weight (N=176)	<1500	14	6 (42.9)	8 (57.1)
	1500 – 2499	48	28 (58.3)	20 (41.7)
	≥2500	114	99 (86.8)	15 (13.2)
Gestational age by NBS*	<37	83	30 (15.1)	53 (26.6)
(N=199)	≥37	116	84 (42.2)	32 (16.1)
Age at admission (N=299)	<7 days	258	156 (60.5)	102 (39.5)
	≥7days	41	36 (87.8)	5 (12.2)
Resuscitation at birth	Yes	63	30 (47.6)	33 (52.4)
	No	178	119 (66.9)	59 (33.1)

* New Ballard Score

**Maternal socio-demographic, obstetric and medical characteristics**: The majority of the mothers were between 18 and 35 years (284/304, 93.3%) and about half (146/304, 48.0%) were illiterate. Regarding the obstetric characteristics, 44.9% (136/304) of the mothers were primipara; most (291/304, 95.8%) had at least one ANC follow up visit, and the majority delivered at health facilities (284/304, 94.3%). Only very few of the mothers had associated medical illnesses with hypertension, human immunodeficiency virus (HIV), congestive heart failure and diabetes mellitus seen in 10 (3.2%), 2 (0.7%), 2 (0.7%) and 1 (0.3%) respectively (Table 2 and 3).

**Table 2:** Maternal socio-demographic characteristics in neonates admitted to JMC

Variable	Category	Total N (%)	Sepsis, N (%)	No sepsis, N (%)
Maternal age in years (N=298)	<18	6	2 (33.3)	4 (66.7)
	18–35	284	188 (66.2)	96 (33.8)
	>35	8	4 (50.0)	4 (50.0)
Residency (N=281)	Urban	121	80 (66.1)	41 (33.9)
	Rural	160	101 (63.1)	59 (36.9)
Educational status (N=297)	None	111	73 (65.8)	38 (34.2)
	Read and write only	35	26 (74.3)	9 (25.7)
	Primary	60	37 (61.7)	23 (38.3)
	Secondary	50	34 (68.0)	16 (32)
	Above high school	41	23 (56.1)	18 (43.9)
Occupation (N=295)	Housewife	214	141 (65.9)	73 (34.1)
	Self employed	28	22 (78.6)	6 (21.4)
	Government employee	31	19 (61.3)	12 (38.7)
	Farmer	13	6 (46.2)	7 (53.8)
	Other	9	5 (55.6)	4 (44.4)

**Table 3:** Maternal obstetric and medical conditions in neonates admitted to JMC

Variable		Total	Sepsis, N (%)	No sepsis, N (%)	p (95% CI)
Parity (N=303)	1	136	91 (66.9)	45 (33.1)	.15 (.85, 2.81)
	2–4	126	79 (62.7)	47 (37.3)	
	≥5	41	25 (61.0)	16 (39.0)	
Antenatal care (N=304)	Yes	291	185(63.6)	106 (36.4)	.36 (.31, 25.98)
	No	13	10 (76.9)	3 (23.1)	
Urinary tract infection (N=304)	Yes	14	11 (78.6)	3 (21.4)	.87 (.21, 3.81)
	No	290	184 (63.4)	106 (36.6)	
Prolonged rupture of membrane (N=298)	Yes	36	29 (80.6)	7 (19.4)	.09 (.87, 7.81)
	No	262	160 (61.1)	102 (38.9)	
Prolonged labor (N=301)	Yes	42	35 (83.3)	7 (16.7)	.32 (.62, 4.17)
	No	259	158 (61.0)	101 (39.0)	
Fever in the last 7 days before delivery (N=304)	Yes	11	9 (81.8)	2 (18.2)	.45 (.09, 2.83)
	No	293	186 (63.5)	107 (36.5)	
Fever during labor and delivery (N=304)	Yes	19	14 (73.7)	5 (26.3)	.49 (.22, 2.08)
	No	285	181 (63.5)	104 (36.5)	
Foul smelling liquor (N=274)	Yes	9	7 (77.8)	2 (22.2)	.33 (.31, 1.48)
	No	265	165 (62.3)	100 (37.7)	
Chorioamnionitis (N=211)	Yes	10	9 (90.0)	1 (10.0)	.34 (.82, 1.80)
	No	201	122 (60.7)	79 (39.3)	
Meconium stained amniotic fluid (N=218)	Yes	22	14 (4.7)	8 (2.7)	.17 (.16, 1.39)
	No	196	125 (41.8)	71 (23.7)	
Place of delivery (N=301)	Institutional	284	184 (67.8)	100 (32.2)	.78 (.18, 10.04)
	Home	17	10 (58.8)	7 (41.2)	
Mode of delivery (N=299)	SVD	188	114 (38.1)	74 (24.7)	.47 (.70, 2.15)
	CS	104	75 (25.1)	29 (9.7)	
	ID	7	3 (1.0)	4 (1.3)	

SVD: Spontaneous vaginal delivery; CS: Cesarean section; ID: Instrumental delivery

Among maternal obstetric conditions, prolonged labor (more than 24hours) was seen in 13.9% (42/304) whereas prolonged rupture of membrane (more than 18hours) and meconium stained amniotic fluid were reported in 11.9% (36/304) and 7.4% (22/304) of the mothers respectively. Moreover, urinary tract infection (4.5%; 14/304), fever in the last 7 days before delivery (3.6%; 11/304), chorioamnionitis (3.3%; 10/304) and foul-smelling amniotic fluid (3.0%; 9/304) were documented in small proportion of the mothers (Tables 2 and 3).

**Figure 1:**
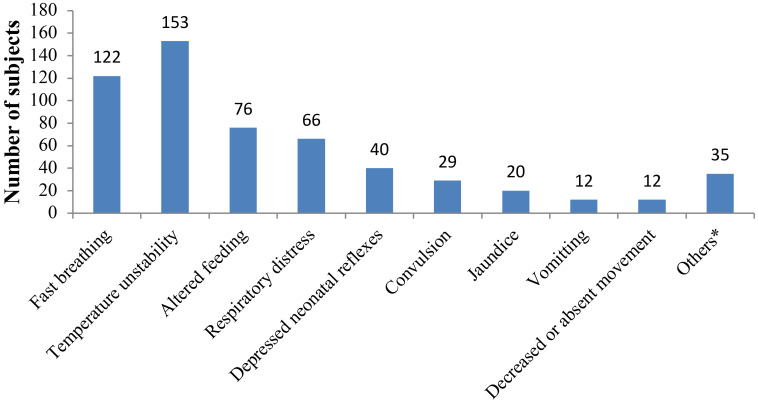
Clinical presentation of neonates admitted with sepsis to NICU of JMC [*Include apnea (7), skin pustules (7), pallor (6), eye discharge (5), abdominal distension (5), umbilical discharge (3), and bulged fontanels (2)]

**Clinical profile of neonates admitted with sepsis:** Out of the 304 neonates included in this study, 195 (64.1%) had sepsis according to the clinical definition, majority (84.1%; 164/195) of them having EONS. The most frequently observed signs and symptoms were fast breathing (62.6%; 122/195), fever (46.7%; 91/195), altered feeding (39.0%, 76/195), respiratory distress (33.8%; 66/195) and hypothermia (31.8%; 62/195) (Figure 1).

**Laboratory investigations**: Microbiological investigations only in the subgroup of neonates with signs of sepsis were conducted and included in this work. Blood culture was done for 96.4% (188/195) of the neonates with suspected sepsis. Of these, 61.2% (115/188) were positive; 109 were bacteria whereas 6 were fungi. In two neonates, multiple organisms were detected on blood culture. *Coagulase Negative Staphylococcus (CONS)* (25.7%; 28/109), *S. aureus* (22.0%; 24/109) and *Klebsiella spp.* (16.5%; 18/109) were the three predominant bacteria isolated. Other gram positive bacteria isolated were *micrococcus spp.* (3/109, 2.8%), *Group B streptococcus* (3/109, 2.8%) and *Listeria monocytogenes* (1/109, 0.9%) whereas the other gram negative bacteria isolated were *E. coli* (2/109, 1.8%), *Enterobacter spp*. (2/109, 1.8%), *Providentia spp*. (2/109, 1.8%), *Proteus mirabilis* (1/109, 0.9%) and *Serratia spp*. (1/109, 0.9%).

Lumbar puncture and CSF culture were performed for 68.2% (133/195) and 72.9% (97/133) of neonates with sepsis respectively. Pleocytosis (white cell count of ≥15cells/mm^3^) was detected in 8.3% (11/133) whereas culture was positive in only 4.1% (4/97). No microorganism was detected on CSF gram stain (microscopy). The organisms isolated from the CSF cultures were *Citrobacter spp. (1), K. pneumoniae* (2) and *Acinetobacter spp. (1)*.

**Treatment given to those with sepsis:** Majority of the newborns with sepsis, 90% (171/190), received combination of ampicillin and gentamicin whereas 16/190 (8.4%) received combination of ceftriaxone and gentamicin. Only few of them received combination of ceftazidime and vancomycin (3/190, 1.6%).

**Figure 2:**
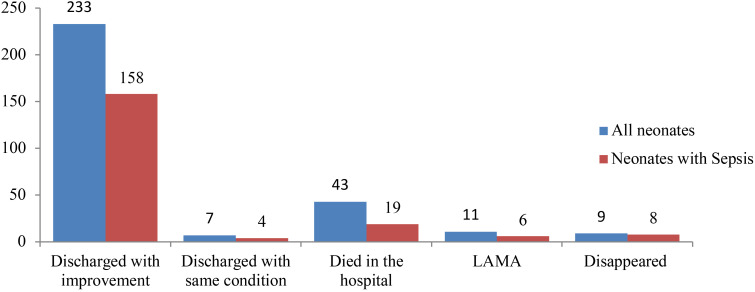
Discharge status of neonates admitted to NICU of JMC LAMA – leave against medical advice

**Clinical outcome of the neonates**: Overall, the majority of the neonates were discharged with improvement (233, 76.9%), whereas 43 (14.2%) died in the hospital. Out of the 43 deaths, 19 (44.2%) had sepsis and 14 of these 19 deaths with sepsis (73.7%) had positive blood culture results. This gives a death rate among those with clinical sepsis 9.7% (19/195) whereas the death rate among the culture confirmed ones is 12.2% (14/115). The death rate among the gram negative isolates is almost 4 times (10/52, 19.2%) higher than that seen in neonates with gram-positive isolates (3/59, 5.1%) (Figure 2).

## Discussion

Almost two thirds (195/304, 64.1%) of the neonates enrolled into this study had clinical sepsis. EONS was 4 times more common than LONS which is similar with several other studies in which EONS was at least 3 times as common as LONS (10,13,15).

The most frequent clinical manifestations seen in our study were similar to findings of a study done in Northern Ethiopia, in which fever, irregular respirations and tachypnea were the three commonest symptoms of illness (18). Similarly, other studies done in other countries have reported respiratory distress, poor feeding and fever as the most frequent symptoms of illness (19–21).

The three most frequently seen obstetric conditions in the neonates with sepsis in this study were prolonged labor, premature rupture of membrane and meconium stained amniotic fluid which is similar to a study done in Southern Ethiopia (22). The frequency of seeing the well known risk factors for neonatal sepsis like maternal urinary tract infection (UTI), fever during pregnancy and short before delivery, chorioamnionitis, and foul smelling amniotic fluid among those with sepsis was very low, each accounting for only <5.0%. This is similar to a study done in Northern Ethiopia where only 4% of the mothers had UTI (18) and a study from Southern Ethiopia where only <5% of the mothers had UTI, chorioamnionitis and foul smelling liquor (22). But it is in contrast to other studies done in Ethiopia which have reported higher rate of maternal fever, UTI/sexually transmitted infections (STIs) and premature rupture of membrane (15,18,23). This difference might be explained by the difference in the study design used, the timing of the studies and the background characteristics of the patient cohort.

Most of the newborns in this study were born in health facilities. A higher proportion of those born in a health facility were diagnosed with sepsis compared to those delivered at home. This is also reported from other studies (18,22). Among the neonates with sepsis, 30 (15.4%) were resuscitated at birth, which is lower than a report from Mekelle, Northern Ethiopia, in which 44.9% of neonates with sepsis had been resuscitated (23) but higher than what has been reported from Felege Hiwot Hospital where only 3.6% of the neonates with sepsis had been resuscitated (18). This might give some clue about the possible additional risk factors of neonatal sepsis, given the fact that most of our health facilities are deprived of the basic infection prevention and patient safety practices and the fact that the predominant organisms in our study are common floras of the health facilities.

Moreover, this finding may also reflect that diagnosis of neonatal sepsis in newborns born at home or low level health facilities are likely to be missed or delayed and only those born at hospitals are detected and treated. As the rate of home delivery remains very high in Ethiopia (24), findings reported in our study and other hospital based studies in Ethiopia may not reflect the real burden of neonatal morbidities in general and neonatal sepsis in particular. This may be one reason why neonatal mortality in LMICs remains very high.

The three predominant bacteria isolated in the current study were *CONS, S. aureus*, and *Klebsiella spp*. which are entirely different from the usual etiologies of neonatal sepsis (*Listeria monocytogenes, Group B streptococcus* and gram negatives like *E. coli*). This finding is similar to a study done in North-western Ethiopia where *S. aureus, CONS* and *K. pneumoniae* were the predominant organisms (15). The result is also in line with other literatures which indicate *Klebsiella spp. S. aureus* and *CONS* to be responsible for neonatal sepsis in developing countries (25). Hence, large, facility and community based studies are required to see the etiologies of neonatal sepsis in order to make the necessary modifications of antimicrobial treatment.

As we tried to highlight above, our study which is limited to a university hospital may not reflect the real burden of neonatal sepsis in Ethiopia because of the fact that most mothers still give birth at home and most institutional deliveries happen at low level healthcare facilities. The other limitation in our study is that we only focused on bacterial etiologies whereas neonatal sepsis could be due to other etiologies including viruses. Furthermore, as we collected only one blood culture, the possibilities of contamination cannot be ruled out. Finally, some of the neonates might have received antimicrobials prior to collection of blood for microbiologic testing which might have affected the blood culture positivity rate and hence, probably the type of microorganisms responsible for sepsis in the study site. Nevertheless, we believe that our finding, along with similar recent studies in the country, may help improve empiric management of neonates with clinical suspicion of sepsis.

In this study, we have demonstrated that majority of the neonates had EONS and were born in a health facility, the latter of which is thought to reduce the burden of neonatal infections. Additionally, we have shown that the bacterial etiologies isolated in our study, predominantly *CONS, S. aureus* and *Klebsiella* spp, differ from reports from both high and low income settings. The fact that these organisms are predominantly nosocomial in origin and that many of the neonates were born in health facilities (the potential sources of these organisms) highlights the importance of infection prevention and control practices of the health facilities, particularly in the labor and delivery units, including the process of neonatal resuscitation. Additionally, continuous surveillance of the etiologies of neonatal sepsis and possible revision of the empiric antimicrobial treatment of neonatal sepsis targeting these commonest etiologies is recommended.

## References

[1] United Nations Inter-agency Group for Child Mortality Estimation (UNIGME) (2018). Levels & Trends in Child Mortality: Report 2019, Estimates developed by the United Nations Inter-agency Group for Child Mortality Estimation.

[2] World Health Organization (2015). Guideline managing possible serious bacterial infection in young infants when referral is not feasible.

[3] Ethiopian Public Health Institute (EPHI) [Ethiopia] and ICF (2019). Ethiopia Mini Demographic and Health Survey 2019: Key Indicators.

[4] Stoll Barbara J, Hansen Nellie, Fanaroff Avroy A, Wright Linda L, Carlo Waldemar A, Ehrenkranz Richard A (2002). Changes in pathogens causing early-onset sepsis in very-low-birth-weight infants. The New England Journal of Medicine.

[5] Ferreira Rachel C, Mello Rosane R, Silva Kátia S (2014). Neonatal sepsis as a risk factor for neurodevelopmental changes in preterm infants with very low birth weight. Jornal de Pediatr.

[6] Dammann Olaf, Kuban Karl C K, Leviton Alan (2002). Perinatal Infection, Fetal Inflammatory Response, White Matter Damage, And Cognitive Limitations In Children Born Preterm. Mental Retardation And Developmental Disabilities Research Reviews.

[7] Zea-Vera Alonso, Ochoa Theresa J (2015). Challenges in the diagnosis and management of neonatal sepsis. Journal of Tropical Pediatrics.

[8] Seale AC, Obiero CW, Berkley JA (2015). Rational development of guidelines for management of neonatal sepsis in developing countries. Curr Opin Infect Dis.

[9] Shrestha S, Adhikari N, Rai B K, Shreepaili A (2010). Antibiotic resistance pattern of bacterial isolates in neonatal care unit. Journal of the Nepal Medical Association.

[10] Pokhrel Bhishma, Koirala Tapendra, Shah Ganesh, Joshi Suchita, Baral Pinky (2018). Bacteriological profile and antibiotic susceptibility of neonatal sepsis in neonatal intensive care unit of a tertiary hospital in Nepal. BMC Pediatrics.

[11] Ghotaslou R, Ghorashi Z, Nahaei MR (2007). Klebsiella pneumoniae in neonatal sepsis: a 3-year-study in the pediatric hospital of Tabriz, Iran. Japanese Journal of Infectious Diseases.

[12] Ghotaslou R, Ghorashi Z, Nahaei MR (2007). Klebsiella pneumoniae in neonatal sepsis: a 3-year-study in the pediatric hospital of Tabriz, Iran. Japanese Journal of Infectious Diseases.

[13] Huynh Bich-Tram, Padget Michael, Garin Benoit, Herindrainy Perlinot, Kermorvant-Duchemin Elsa, Watier Laurence (2015). Burden of bacterial resistance among neonatal infections in low income countries: how convincing is the epidemiological evidence?. BMC Infectious Disease.

[14] Jyothi P, Basavaraj Metri C, Basavaraj Peerapur V (2013). Bacteriological profile of neonatal septicemia and antibiotic susceptibility pattern of the isolates. Journal of Natural Science, Biology and Medicine.

[15] Paolucci Michela, Landini Maria Paola, Sambri Vittorio (2012). How can the Microbiologist Help in Diagnosing Neonatal Sepsis?. International Journal of Pediatrics.

[16] G/eyesus Tsehaynesh, Moges Feleke, Eshetie Setegn, Yeshitela Biruk, Abate Ebba (2017). Bacterial etiologic agents causing neonatal sepsis and associated risk factors in Gondar, Northwest Ethiopia. BMC Pediatrics.

[17] Sorsa Abebe (2019). Epidemiology of Neonatal Sepsis and Associated Factors Implicated: Observational Study at Neonatal Intensive Care Unit of Arsi University Teaching and Referral Hospital, South East Ethiopia. Ethiop J Health Sci.

[18] Cheesbrough M (2006). District laboratory practice in tropical countries.

[19] Tewabe Tilahun, Mohammed Seida, Tilahun Yibeltal, Melaku Birhanie, Fenta Mequanint, Dagnaw Tsigiereda (2017). Clinical outcome and risk factors of neonatal sepsis among neonates in Felege Hiwot referral Hospital, Bahir Dar, Amhara Regional State, North West Ethiopia 2016: a retrospective chart review. BMC Research Notes.

[20] El-Din Eman M Shehab Rabie, Adel El-Sokkary Mohamed M, Bassiouny Mohamed Reda, Hassan Ramadan (2015). Epidemiology of Neonatal Sepsis and Implicated Pathogens: A Study from Egypt. BioMed Research International.

[21] Arowosegbe Adediwura O, Ojo David A, Dedeke Iyabode O, Shittu Olufunke B, Akingbade Olusola A (2017). Neonatal sepsis in a Nigerian Tertiary Hospital: Clinical features, clinical outcome, aetiology and antibiotic susceptibility pattern. Southern African Journal of Infectious Diseases.

[22] Mohsen Lamiaa, Ramy Nermin, Saied Dalia, Akmal Dina, Salama Niveen, Abdel Haleim Mona M (2017). Emerging antimicrobial resistance in early and lateonset neonatal sepsis. Antimicrobial Resistance and Infection Control.

[23] Getabelew Aytenew, Aman Mihret, Fantaye Endashaw, Yeheyis Tomas (2018). Prevalence of Neonatal Sepsis and Associated Factors among Neonates in Neonatal Intensive Care Unit at Selected Governmental Hospitals in Shashemene Town, Oromia Regional State, Ethiopia, 2017. Hindawi International Journal of Pediatrics.

[24] Gebremedhin Destaalem, Berhe Haftu, Gebrekirstos Kahsu (2016). Risk Factors for Neonatal Sepsis in Public Hospitals of Mekelle City, North Ethiopia, 2015: Unmatched Case Control Study. PLOS ONE.

[25] Chernet Ayele Gebeyehu, Dumga Kassahun Trueha, Cherie Kebadu Tadesse (2019). Home Delivery Practices and Associated Factors in Ethiopia. J Reprod Infertil.

